# Supplementary material for: Differential methods for assessing sensitivity in biological models
Source: PLoS Comput Biol. 2022 Jun 13;18(6):e1009598. doi: 10.1371/journal.pcbi.1009598 (PMC9232177; doi:10.1371/journal.pcbi.1009598)
Supplement: S1 Appendix — (DOCX) [file pcbi.1009598.s001.docx]

# S1 Appendix: Derivation of Second Derivative Complex Perturbation Method

To prove the formulas for approximating partial derivatives stated in the text, we first note that any analytic function $f\left( \mathbf{z} \right)$ of several variables can be expanded in a locally convergent power series about every point $\mathbf{z}$ of its open domain of definition. If we choose a real direction vector $\mathbf{v}$, then the function $g\left( w \right)=f\left( \mathbf{z}+w\mathbf{v} \right)$ is locally analytic in the complex plane $\{\mathbf{z}+w\mathbf{v}:w\mathbb{\in C\}}$ and can be expanded in a power series around $w=0$. Thus,

Equation A1:

$$\begin{matrix} g\left( w \right) & = & \sum_{j=0}^{d} \frac{d^{j}}{dw^{j}}g\left( \mathbf{0} \right)\frac{w^{j}}{j!}+O\left( \left| w \right|^{d+1} \right) \end{matrix}$$

for any integer $d\geq0$. Now consider the setting where $\mathbf{z}$ has real components. If $f\left( \mathbf{z} \right)$ is real valued, then the derivatives $\frac{d^{j}}{dw^{j}}g\left( \mathbf{0} \right)$ will be real as well. One can exploit this fact in approximating the derivatives. For example, if $w=i$, then $w^{j}$ rotates among the four values $1$, $i$, $-1$, and $-i$. Because the terms of the expansion (eq. A1) alternate between real and imaginary values, the first partial derivative formula

$$\begin{matrix} g'\left( \beta\right) & = & \frac{\text{Imag }g\left( \beta+\Delta i \right)}{\Delta}+O\left( \Delta^{2} \right) \end{matrix}$$

holds. For the choice $w=e^{\pi i/4}$, the powers $w^{d}$ rotate among the eight values $1$, $e^{\pi i/4}$, $i$, $ie^{\pi i/4}$, $-1$, $-e^{\pi i/4}$, $-i$, and $-ie^{\pi i/4}$. The powers $\left( -w \right)^{j}=\left( -1 \right)^{j}w^{j}$ agree in this regard except for sign. Hence, the terms in the expansion of the sum

$$\begin{matrix} g\left[ \mathbf{x}+e^{\pi i/4}\Delta\left( \mathbf{e}_{j}+\mathbf{e}_{k} \right) \right]+g\left[ \mathbf{x}-e^{\pi i/4}\Delta\left( \mathbf{e}_{j}+\mathbf{e}_{k} \right) \right] \end{matrix}$$

alternately cancel and reinforce. Thus, the first five terms of the expansion are real, 0, imaginary, 0, real, 0. It follows that the imaginary part of the sum is accurate to order $O\left( \Delta^{6} \right)$ and that the approximations

$$\begin{matrix} \frac{\partial^{2}}{\partial\beta_{j}^{2}}g\left( \boldsymbol{\beta} \right) & = & \frac{\text{Imag }\left[ g\left( \boldsymbol{\beta}+e^{\pi i/4}\Delta\mathbf{e}_{j} \right)+g\left( \boldsymbol{\beta}-e^{\pi i/4}\Delta\mathbf{e}_{j} \right) \right]}{\Delta^{2}}+O\left( \Delta^{4} \right) \end{matrix}$$

and

$$\begin{matrix} & & \frac{\text{Imag }\{g\left[ \mathbf{x}+e^{\pi i/4}\Delta\left( \mathbf{e}_{j}+\mathbf{e}_{k} \right) \right]+g\left[ \mathbf{x}-e^{\pi i/4}\Delta\left( \mathbf{e}_{j}+\mathbf{e}_{k} \right) \right]\}}{\Delta^{2}} \\ & = & \left[ \left( \mathbf{e}_{j}+\mathbf{e}_{k} \right) \right]^{\top}d^{2}g\left( \mathbf{x} \right)\left[ \left( \mathbf{e}_{j}+\mathbf{e}_{k} \right) \right]+O\left( \Delta^{4} \right) \\ & = & \frac{\partial^{2}}{\partial\beta_{j}^{2}}g\left( \boldsymbol{\beta} \right)+\frac{\partial^{2}}{\partial\beta_{k}^{2}}g\left( \boldsymbol{\beta} \right)+2\frac{\partial^{2}}{\partial\beta_{j}\partial\beta_{k}}g\left( \boldsymbol{\beta} \right)+O\left( \Delta^{4} \right) \end{matrix}$$

are accurate to order $O\left( \Delta^{4} \right)$.
