# Supplementary material for: Differential methods for assessing sensitivity in biological models
Source: PLoS Comput Biol. 2022 Jun 13;18(6):e1009598. doi: 10.1371/journal.pcbi.1009598 (PMC9232177; doi:10.1371/journal.pcbi.1009598)
Supplement: S2 Appendix — (DOCX) [file pcbi.1009598.s002.docx]

**S2 Appendix: Additional Models**

## The Mammalian Cell Cycle Model

The Mammalian Cell Cycle Model presented in section 4 of this manuscript is a model originally described in [1] and simplified in the BioModels [2] database. This system describes the interaction of cyclin-dependent kinases (Cdk) with Cdk inhibitors, growth factors, and other proteins that regulate the development of mammalian cells. The model includes characteristics such as cell cycling, tumor repressor initiated progression control, and cell cycle completion. The ODE system representing the model is

$$\begin{matrix} \frac{dpRBc1}{dt} & =kpc1*pRB*E2F \\ \frac{dpRBc2}{dt} & =kpc3*pRBp*E2F \\ \frac{dCd}{dt} & =kcd1*AP1+kdecom1*Mdi \\ & -kcom1*Cd*\left( Cdk4_{tot}-\left( Mdi+Md+Mdp27 \right) \right) \\ & +kcd2*E2F*\frac{Ki7}{Ki7+pRB}*\frac{Ki8}{Ki8+pRBp} \\ \frac{dMdi}{dt} & =Vm2d*\frac{Md}{k2d+Md}+2*kcom1*Cd*\left( Cdk4_{tot}-\left( Mdi+Md+Mdp27 \right) \right) \\ \frac{dMd}{dt} & =Vm1d*\frac{Mdi}{k1d+Mdi}+kcom1*Cd*\left( Cdk4_{tot}-\left( Mdi+Md+Mdp27 \right) \right) \\ \frac{dpRB}{dt} & =kcd2*E2F*\frac{Ki7}{Ki7+pRB}*\frac{Ki8}{Ki8+pRBp} \\ \frac{dE2F}{dt} & =kcd2*E2F*\frac{Ki7}{Ki7+pRB}*\frac{Ki8}{Ki8+pRBp} \\ \frac{dpRBp}{dt} & =kcd2*E2F*\frac{Ki7}{Ki7+pRB}*\frac{Ki8}{Ki8+pRBp} \\ \frac{dAP1}{dt} & =kcd1*AP1 \\ \frac{dp27}{dt} & =0 \\ \frac{dMdp27}{dt} & =kc1*Md*p27+kcom1*cd*\left( Cdk4_{tot}-\left( Mdi+Md+Mdp27 \right) \right) \end{matrix}$$

The initial values of each compartment and parameter are defined as

| Compartment | Initial Value ($\mu$ mol) | Parameter | Value |
| --- | --- | --- | --- |
| pRBc1 | 0.1 | kpc1 | 0.05 |
| pRBc2 | 0.05 | kpc3 | 0.025 |
| Cd | 0.01 | kcd1 | 0.4 |
| Mdi | 0.01 | Ki8 | 2.0 |
| Md | 0.01 | Ki7 | 0.1 |
| pRB | 0.0 | kcd2 | 0.005 |
| E2F | 0.0 | kdecom1 | 0.1 |
| pRBp | 0.0 | k2d | 0.1 |
| AP1 | 0.0 | Cdk$4_{\text{tot}}$ | 1.5 |
| p27 | 0.0 | kcom1 | 0.175 |
| Mdp27 | 0.0 | Vm2d | 0.2 |
| – | – | k1d | 0.1 |
| – | – | Vm1d | 1.0 |
| – | – | kc1 | 0.15 |

##

## The ROBER Model

The ROBER Model presented in section 4 of this manuscript refers to the auto-catalytic chemical reaction of Robertson as described in [3]. This model is often used as an example of a classic stiff ODE system encountered in biology. The ODE system this model represents is

$$\begin{matrix} \frac{dx_{1}}{dt} & = & -p_{1}x_{1}+p_{3}x_{2}x_{3} \\ \frac{dx_{2}}{dt} & = & p_{1}x_{1}-p_{2}x_{2}^{2}-p_{3}x_{2}x_{3} \\ \frac{dx_{3}}{dt} & = & p_{2}x_{2}^{2} \end{matrix}$$

The initial values of each compartment and parameter are defined as

| Compartment | Initial Value | Parameter | Value |
| --- | --- | --- | --- |
| $x_{1}$ | $1.0$ | $p_{1}$ | $4\times{10}^{-2}$ |
| $x_{2}$ | $0.0$ | $p_{1}$ | $3\times{10}^{7}$ |
| $x_{3}$ | $0.0$ | $p_{1}$ | $1\times{10}^{4}$ |

**References**

1 Gérard C, Goldbeter A. Temporal self-organization of the cyclin/Cdk network driving the mammalian cell cycle. Proceedings of the National Academy of Sciences. 2009 Dec 22;106(51):21643-8.

2 Malik-Sheriff RS, Glont M, Nguyen TV, Tiwari K, Roberts MG, Xavier A, Vu MT, Men J, Maire M, Kananathan S, Fairbanks EL. BioModels—15 years of sharing computational models in life science. Nucleic acids research. 2020 Jan 8;48(D1):D407-15.

3 Robertson HH. The Solution of a Set of Reaction Rate Equations Numerical Analysis.
