# Supplementary material for: Differential methods for assessing sensitivity in biological models
Source: PLoS Comput Biol. 2022 Jun 13;18(6):e1009598. doi: 10.1371/journal.pcbi.1009598 (PMC9232177; doi:10.1371/journal.pcbi.1009598)
Supplement: S3 Appendix — (DOCX) [file pcbi.1009598.s003.docx]

# S3 Appendix: Sensitivity of Linear Systems

The simplest dynamical models are governed by the linear constant coefficient differential equation $\frac{d}{dt}\mathbf{x}\left( t \right)=\mathbf{A}\left( \boldsymbol{\beta} \right)\mathbf{x}\left( t \right)$ with solution $\mathbf{x}\left( t \right)=e^{t\mathbf{A}\left( \boldsymbol{\beta} \right)}\mathbf{x}_{0}$, where $\mathbf{A}\left( \boldsymbol{\beta} \right)$ is any function differentiable in its parameters $\boldsymbol{\beta}$ and constant in $t$ . The directional derivative of the matrix exponential $e^{\mathbf{B}}$ in the direction $\mathbf{V}$ can be represented by the integral

$$\begin{matrix} d_{\mathbf{V}}e^{\mathbf{B}} & = & \int_{0}^{1} e^{s\mathbf{B}}\mathbf{V}e^{\left( 1-s \right)\mathbf{B}}ds . \end{matrix}$$

A simple proof of this fact appears in Example 3.2.2 of reference [1]. Setting $\mathbf{B}=t\mathbf{A}\left( \boldsymbol{\beta} \right)$ and applying the chain rule leads to the partial derivative

$$\begin{matrix} \frac{\partial}{\partial\beta_{j}}e^{t\mathbf{A}\left( \boldsymbol{\beta} \right)}\mathbf{x}\left( 0 \right) & = & \int_{0}^{1} e^{st\mathbf{A}\left( \boldsymbol{\beta} \right)}t\frac{\partial}{\partial\beta_{j}}\mathbf{A}\left( \boldsymbol{\beta} \right)e^{\left( 1-s \right)t\mathbf{A}\left( \boldsymbol{\beta} \right)}ds \mathbf{x}\left( 0 \right) \\ & = & \int_{0}^{t} e^{s\mathbf{A}\left( \boldsymbol{\beta} \right)}\frac{\partial}{\partial\beta_{j}}\mathbf{A}\left( \boldsymbol{\beta} \right)e^{\left( t-s \right)\mathbf{A}\left( \boldsymbol{\beta} \right)}ds \mathbf{x}\left( 0 \right), \end{matrix}$$

which can be laboriously evaluated by numerical integration. Simplification into a sum of exponentials is possible if $\mathbf{A}\left( \boldsymbol{\beta} \right)$ is uniformly diagonalizable across all $\boldsymbol{\beta}$ [2].

In practice, it is simpler to differentiate the original ODE with respect to $\beta_{j}$, interchange the order of differentiation, and numerically integrate the system

$$\begin{matrix} \frac{d}{dt}\frac{\partial}{\partial\beta_{j}}\mathbf{x}\left( t,\boldsymbol{\beta} \right) & = & \frac{\partial}{\partial\beta_{j}}\mathbf{A}\left( \boldsymbol{\beta} \right)\mathbf{x}\left( t,\boldsymbol{\beta} \right)+\mathbf{A}\left( \boldsymbol{\beta} \right)\frac{\partial}{\partial\beta_{j}}\mathbf{x}\left( t,\boldsymbol{\beta} \right) \end{matrix}$$

from $0$ to some final value of $t$. The initial condition $\mathbf{x}\left( 0,\boldsymbol{\beta} \right)=\mathbf{x}\left( 0 \right)$ remains intact, and the new condition $\nabla_{\boldsymbol{\beta}}\mathbf{x}\left( 0,\boldsymbol{\beta} \right)=0$ is added.

**References**

1 Lange K. MM optimization algorithms. Society for Industrial and Applied Mathematics; 2016 Jul 5.

2 Dorman KS, Sinsheimer JS, Lange K. In the garden of branching processes. SIAM review. 2004;46(2):202-29.
