# Supplementary material for: Differential methods for assessing sensitivity in biological models
Source: PLoS Comput Biol. 2022 Jun 13;18(6):e1009598. doi: 10.1371/journal.pcbi.1009598 (PMC9232177; doi:10.1371/journal.pcbi.1009598)
Supplement: Attachment — Submitted filename: reviewresponse_DifferentialSensitivity.pdf [file pcbi.1009598.s005.pdf]

To Whom It May Concern,

The following document contains summary responses to each reviewer, as well as detailed responses to reviewer comments, and excerpts from changes to the manuscript. Reviewers may note that three substantial changes have been made to the manuscript after receiving these reviews –

(1) Readability has been improved. We have standardized our notation, added figure and table captions, changed our plotting style, and added additional explanatory information, in addition to other aesthetic and readability suggestions by reviewers.

(2) We have added more information about how this manuscript adds to the broader literature of differential sensitivity analysis. We have added a small literature review of parameter estimation methods to the Introduction section and an examination of the benefits and limitations of our analysis in the Discussion section.

(3) We have made substantial changes to our Benchmarking section. In addition to reorganizing the section for readability, we have added an additional biological model to demonstrate the performance of these differential sensitivity methods on stiff ODEs. Additionally, we have added experiments that test (a) the convergence of the forward, adjoint, and complex perturbation methods towards the true derivatives and (b) the relationship between speed and accuracy in the forward and complex perturbation methods in order to facilitate a discussion of the tradeoffs between these elements of computation.

Thank you for the opportunity to improve our manuscript by implementing the suggestions provided in these reviews.

Sincerely,
Rachel Mester

Reviewer #1:

<u>Summary for Reviewer #1</u>

Overall, reviewer 1 did not request major structural changes or additional numerical experiments. However, reviewer 1 did provide several helpful suggestions to enhance the clarity of the manuscript as well as to make it more clear how much of an impact this manuscript can have on parameter estimation. We have implemented all of these suggestions, and the reviewer may be most interested in a paragraph added to the introduction that serves as both a short literature review of methods for parameter estimation as well as an explanation for how differential sensitivity can aid in that process.

<u>Responses to Individual Comments for Reviewer #1</u>

In the Introduction part, it would be even more motivating to write about how sensitivity may determine the quality of parameter estimation results, e.g. in the context of structural and/or practical identifiability.

We have added a paragraph to the introduction that introduces the topic of parameter estimation, its importance to the field of biological modeling, and the role that differential sensitivity plays in determining parameter estimation. This paragraph serves as a partial literature review of common differential sensitivity methods and as a reminder to the reader that a pertinent context for differential sensitivity is in identifying parameter values. This paragraph is reproduced below for convenience:

Exerpt 1

Additionally, parameter estimation for model fitting usually involves differential sensitivity through maximum likelihood or least squares criteria. These optimization techniques depend heavily on gradients and Hessians with respect to parameters. While some parameter estimation methods rely on Bayesian computational techniques [1] rather than gradients, these techniques tend to scale poorly as the number of model parameters increases. A common way to alleviate the poor scaling of Bayesian inference is Hamiltonian Monte Carlo [2], which itself requires gradient calculations. Techniques for assessing sensitivity of stochastic models often rely on the gradient-dependent Fisher information matrix of the model, which is the basis for a variety of multi-step local sensitivity analysis techniques for discrete stochastic models [3].

Calculation of gradients and Hessians of a model can also be important in other steps of the scientific process. For example, iterative model development [4] involves utilizing the Fisher information matrix in order to inform experimental design. Extended Kalman filtering [5] incorporates differential sensitivity into model construction. Regardless of the method, parameter estimation is an important step in fitting a biological model, and the success of the step strongly impacts the ultimate utility of the model. Understanding the uses and limitations of differential sensitivity can aid in determining the identifiability of model parameters, how sensitive they are to experimental error or measurement noise, and the overall importance of their existence in the model. Finally, it is worth noting that while local sensitivity analysis is the focus of this manuscript, global sensitivity analysis often relies on local differential sensitivity estimates to inform optimal stepsizes in regional searching [6] or to resolve inconsistencies that arise when local sensitivity is non-monotonic [7].

[1] Liepe J, Kirk P, Filippi S et al. A framework for parameter estimation and model selection from experimental data in systems biology using approximate Bayesian computation. Nat Protoc. 2014; 9:439–456.

[2] Neal, RM. MCMC using Hamiltonian dynamics. Handbook of markov chain monte carlo.2011.

[3] Gunawan R, Cao Y, Petzold L, Doyle FJ. Sensitivity Analysis of Discrete Stochastic Systems. Biophysical Journal. 2005; 88:4:2530-2540.

[4] Gadkar KG, Gunawan R,Doyle FJ. Iterative approach to model identification of biological networks. BMC Bioinformatics. 2005; 6:155.

[5] Lillacci G, Khammash M. Parameter Estimation and Model Selection in Computational Biology. PLOS Computational Biology. 2010.

[6] Balsa-Canto E, Banga JR. AMIGO, a toolbox for advanced model identification in systems biology using global optimization. Bioinformatics. 2011; 27:162311–2313.

[7] Marino S, Hogue IB, Ray CJ, Kirschner DE, A methodology for performing global uncertainty and sensitivity analysis in systems biology. Journal of Theoretical Biology. 2008; 254:1:178-196.

I'd suggest structuring Section 2 into subsections corresponding to the different methods described.

As suggested, we have split up Section 2 into subsections, titled "Forward Methods", "Adjoint Methods", "Complex Perturbation Methods", and "Automatic Differentiation" that describe their respective methods.

Various notes about typos.

We have fixed all identified typos, as well as thoroughly read through the manuscript to identify and correct any additional typos not identified by the reviewers.

I'd suggest changing superscript t for the notation of transposed to something else, e.g. to superscript T. Otherwise it can be confused with the time argument.

Thank you for this suggestion; we have changed all transpose notation to ^{T} in order to enhance clarity.

Please characterize the parameter dependence of matrix A on β in the beginning of Section 2.

We have replaced references to A with references to $A(\beta)$ in order to enhance clarity. Additionally, we have added the following characterization: "$A(\beta)$ is any function differentiable in its parameters β and constant in t."

What is 'original' in Fig. 4?

We have added a caption to Figure 4 to enhance readability of this figure, as well as renamed the four trajectories displayed to "original trajectory, "trajectory with perturbed parameters", "first order prediction", and "second order prediction". The following is from the caption for Figure 4, where (5) and (6) are the equations for the second and first order Taylor approximations.

> Results depend on the SIR model with the original parameters from Figure 3 (original trajectory), re-simulating the SIR trajectory after perturbing the parameters by a random amount around 25% (trajectory with perturbed parameters), approximating the trajectory based on the linear expansion (6) and the first derivative calculated with the complex perturbation method (first order prediction), and approximating the trajectory based on the quadratic expansion (5) and the first and

> second derivatives calculated with the complex perturbation method (second order prediction).

Does irreducibility of the branching process mean that the transition graph is strongly connected?

Yes, irreducibility of the branching process means that the transition graph is strongly connected. We have added the following sentence describing this alternative explanation to the manuscript:

> Equivalently, the process is irreducible when the graph representing transition probabilities is strongly connected.

Reviewer #2:

<u>Summary for Reviewer #2</u>

Overall, reviewer 2 suggested that we improve clarity of which results are novel, and to emphasize more how the benchmarking section is applicable to biologists. Additionally, reviewer 2 requested that we improve the quality of our figures and suggested some improvements to the text. We have made some substantial changes to parts of this manuscript in order to demonstrate biological applicability and also to improve clarity and visual appeal.

The following changes may be of most interest to reviewer 2:

(1) We have added a paragraph to the introduction that introduces the topic of parameter estimation, its importance to the field of biological modeling, and the role that differential sensitivity plays in determining parameter estimation. This paragraph serves as a partial literature review of common differential sensitivity methods and as a reminder to the reader that a pertinent context for differential sensitivity is in identifying parameter values. This paragraph is reproduced in this document for convenience, see <u>*Excerpt 1*</u> *(Additionally, parameter estimation for model fitting…)*

(2) We have restructured our benchmarking section (section 4 of the manuscript) so that our outcomes are more clear and so that the impact that these results may have on biologists is more forthcoming. We now have the following benchmarking sections, each with their own figures and tables: (a) Accuracy, (b) Speed vs Accuracy, (c) Speed, and (d) Prediction Accuracy. Here, we demonstrate (a) which methods can be relied upon to calculate accurate gradients for which types of models, (b) how to assess the tradeoff between speed and accuracy when choosing a tolerance level for the algorithm, (c) what the overall speed comparison is of each of these methods on a variety of representative biological models, and (d) how well each model can be predicted using first and second order Taylor approximations.

(3) We have reformatted all figures using the PyPlot package with custom font sizes, colors, linestyles, and formatted axes. We have broken up tables into more manageable pieces that match with the benchmarking subsections mentioned in point (2). Additionally, we have added captions to all figures and tables with detailed descriptions of how we arrived at our results and any new language or abbreviations that are used.

Responses to Individual Comments for Reviewer #2

The authors attempt to provide a brief overview of adjoint computation methods and include a novel finite difference Hessian that makes use of the complex plane. This section could benefit from adaptation towards people from less mathematical and more biological background. Formulas should be accompanied with geometric intuition or even figures, perhaps with some biological examples of when forward-mode vs reverse-mode autodiff are appropriate? The text needs more work to match mathematical symbols with those that appear in code and text. The narrative is not streamlined yet. At this stage it reads as a stream of conciousness with inline math and code. It is difficult to tell which formulas to pay attention to.

We have reorganized some of the material into more distinct sections that more clearly indicate which methods we are discussing at which time, which we hope will alleviate the feeling of "stream of consciousness" to which you refer. We have also provided an entire section of biological examples (section 3) and a section that attempts to answer questions about when each method is appropriate (section 4). Ideally, these sections are separate from the mathematical methods sections so that an interested reader may skip to the sections of most interest.

it does not add any value to introduce this if it is dropped immediately for the general non-linear case on the next page

While the linear case is only used briefly in this manuscript, we believe it provides an essential introduction to the topic of methods for differential sensitivity. It is common for biologists to be familiar with the topic of dynamical systems and study of differential sensitivity of linear systems through study of models which can be represented as matrices and decomposed into (eigenvector, eigenvalue) pairs which can then be studied for stability. Thus, mention of this topic provides an essential bridge from the realm of methods that are potentially more familiar to biologists to the realm of numerical and direct differentiation methods which may not be as familiar to our audience. Including this topic allows us to show the relative flexibility and ease of use of the differential sensitivity methods we introduce, which would be more difficult without the familiar use case for initial comparison.

Functions need clearer definitions in the form F: R^N -> R^N. Parameters beta live in R^M. Jacobians should be stated as being rectangular N x M or square N x N. The Hessian is a third-order tensor. A bit of geometric intuition when introducing these concepts would help readability….constant interchange of notation hurt readability. I recommend introducing forward and adjoint methods with notation found in Giles, M. 2008. "Collected matrix derivative results for forward and reverse mode automatic differentiation"

We have made efforts to condense our notation into a smaller number of items for the reader to consider, and to keep our notation constant with how ideas are first introduced in the manuscript. However, we have elected to leave out geometric interpretations of these methods due to our focus on numerical and analytic techniques and the requirement for additional notation that geometric arguments would require.

scaling O(Delta) missing

In this case, Δ does not have an inherent scaling and thus it is up to the biologist to determine which scale to use for this parameter. We indicate this choice at the end of the paragraph which contains this sentence. The section on prediction accuracy in section 4 may address some questions as to how to properly scale this parameter for each model, but we acknowledge that it is a difficult problem and likely the best solutions will be adapted to the particular use case at hand.

It is not clear which central difference approximation is referred to here and in practice (especially in Julia) this tends not to be a computational bottleneck. I recommend having a look at FiniteDifferences.jl for benchmarks.

We have addressed this concern by being more clear about which central difference approximation we are referring to. Additionally, while it is beyond the scope of this manuscript to benchmark particular finite difference packages (as opposed to broader differential sensitivity packages), we do note that this is a computation time versus accuracy tradeoff, and that computation time may not be an issue in the case of a small model. Please see the following change to the manuscript in this section:

> Round-off errors can be ameliorated by using central differences
> $$\frac{x\left(t,\beta+\frac{\Delta}{2}v\right)-x\left(t,\beta-\frac{\Delta}{2}\right)}{\Delta}$$
> rather than forward differences. However, the central difference method requires twice the number of computations as the forward difference method. Thus, the choice of a difference method depends on prioritization of accuracy versus computational efficiency. In small models, computational efficiency may be less of a priority, in which case central difference methods are preferred.

take advantage of unicode in Julia and use the Delta symbol so you do not have to introduce d.... the names p1 p2 ... p5 need to be renamed to kappa1, kappa2 ... gamma from the text. Poor effort was made to match up code and text. Please use unicode greek symbols in Julia to aid readability… so much inline math hurts readability… more readable if we get assignment df[:,j] = imag(fj) / d… Purely numerically → using finite differences

Thank you for these great readability suggestions! We have replaced references to Greek letters with their actual symbols, as well as replaced parameter numbers with their names in the code and figures in this manuscript. Additionally, we have reduced the inline math to a minimum by putting equations on separate lines with equation formatting, and replaced our reference to "purely numerically" to the more specific reference to finite differences. For your coding suggestion, we have taken it into consideration. However, we think that concatenating

differentials as column vectors (our current code) is results in a more readable output, since it is generally true in biological models that the time steps are more numerous than the number of parameters in the model. Thus, including the differential of each parameter results in a table likely to take advantage of vertical scrolling, which is preferrable to horizontal scrolling.

Note here that differentiating through operations of algorithms with autodiff requires understanding the details of the algorithm and possibly even patching missing methods for dual numbers / adjoints. Chain rules defined to bypass differentiating through algorithms allows us to treat the algorithms as a black box. This is one of the main lessons of Chen, G. 2019. "Neural Ordinary Differential Equations"

Thank you for pointing out this resource on automatic differentiation. Since automatic differentiation has been implemented in many packages (for example, ForwardDiff.jl, which utilize in this manuscript), we elect to skip over some of the details regarding chain rule definition. Since implementation differs widely by package, level of detail will vary by application, and in many cases biologists will be able to use these implementations "out of the box", we think that a deeper dive into this topic would not benefit our audience enough to merit the substantial detour addressing it would require.

the figures use the default Plots.jl styling which is not ideal for publication-quality figures. I would recommend exporting PDFs with larger axis title fontsizes….what is the scale being show? persumably this shows the finite difference method results for d(x,y)/d(k1,k2).

Thank you for these great suggestions to improve our figures. We have switched to the PyPlot() backend for plotting, as well as added larger axes titles, figure titles, and legends. Additionally, we have added captions to each figure that thoroughly explain what each figure represents. For example, in Figure 1 (where this comment was added), the scale being shown is actual numbers of cancer and immune cells estimated by numerically solving the equations at the different parameter values on the x and y axes.

a lot more work needs to be done to draw the relationship between Figure 1 and 2. What does it mean to see sensititvities d(x,y)/dp along the whole trajectory rather than just at the end point?

To address this question, we have added the following to the Figure 2 discussion in the manuscript

> Figure 2 reinforces the conclusions drawn from the heatmaps, but more clearly and quantitatively. Additionally, differential sensitivity allows for the assessment of the sensitivity over the course of time, rather than just at a single time or small set of times. For example, the sensitivity of x with respect to γ in this model exhibits both large positive and large negative values over the course of time. Measured as the difference in x caused by a difference in γ at our end-time $t = 1000$, these effects tend to cancel each other out and fail to communicate the impact of the parameter γ on x in the intermediate times. In brief, the scaled sensitivity of cancer cells $x(t)$ is much more dependent on carrying capacity γ later in the simulation, while the model sensitivity to birth rate ρ is most pronounced around the earlier time $t = 200$.

no discussion on this figure? missed opportunity to say that the parameters are most sensitive in the early days of the pandemic.

To address this concern, we have added the following sentence to our discussion of Figure 3 in the manuscript:

> Figure 3 captures not only the pronounced parameter sensitivity early on in the pandemic, but also the symmetry between the δ and η parameters.

bit of an absurd statement

To address this concern, we have changed our statement that the parameters capture the American experience to something more specific and objective:

> Our parameter choices roughly capture measurements for the COVID-19 virus from early in the pandemic [8,9].
>
> [8] Toda, AA. Susceptible-infected (SIR) dynamics of COVID-19 and economic impact. arXiv preprint. 2020; 2003.11221
>
> [9] Zhou F et al. Clinical course and risk factors for mortality of adult inpatients with COVID-19 in Wuhan, China: a retrospective cohort study. The Lancet. 2020.

This section outlines an example of how to apply differential sensitivity to a markov chain. This is probably the most interesting example and there may be something novel in the authors approach to disease constact networks. The quality of previous sections alone unforntunately warrent rejection of this paper.

We have addressed the concerns indicated in sections 1, 2, and 3, as well as completely reorganized section 4 to emphasize the novel contributions of this manuscript, which (to the authors knowledge) include the benchmarking, application of the complex perturbation method to stochastic and ODE systems, and the mixed second partial complex perturbation method.

such vocabulary alienates your audience. Perhaps this solution is not obvious to all? Inclusive language avoids the use of "clearly", "trivially" "easy to see that" etc.

Thank you for helping us adopt more inclusive language! We have eliminated all uses of the words “obvious”, “trivial”, and “easy” from the manuscript, and all cases of “clearly” used in this context.

While tables containing performance benchmarks are typical for machine learning papers, these results are not interesting to biological audiences.

Computation is an essential part of the majority of contemporary biological studies [9]. Due to the prevalence of computation (and thus, usage of computational methods) in biology, life

scientists have a vested interest in these methods and their performance. For example, even in the examples we have in this manuscript (which were chosen due to their being representative of models often used in biology), often the computation time from one method to the next varies by orders of magnitude. Computation time is of particular interest to biologists as they may have less easy access to computing clusters than mathematicians and computer scientists. Furthermore, it is important for biologists to understand which methods converge to accurate solutions in the presence of stochastic and stiff equations, since accurate parameter estimation is key in the application of biology to ecological and health sciences, which are fields with large impacts on daily life.

[9] Computation and biology: a partnership. *Nat Methods* **18,** 695 (2021).

Reviewer #3:

Summary for Reviewer #3

Reviewer #3 noted that while our goals are worthwhile, we would need to add some additional numerical analyses and improve our presentation in order to improve the manuscript. Reviewer #3 may be most interested in the improved format and additional numerical experiments that are now a part of section 4 (the benchmarking section). We now have separate subsections for prediction accuracy and speed, as well as subsections that highlight new experiments on method convergence and the speed vs accuracy tradeoff.

Responses to Individual Comments for Reviewer #3

The metrics used to quantify accuracy do not address the accuracy in the calculation of the local sensitivity (section 4, lines 613-614). Rather, it appears that these metrics quantify how well the local behavior of the model can be approximated by a linear or quadratic Taylor approximation. Still, it leaves open whether the differentials themselves are well-approximated by the numerical methods. The latter seems to be more interesting to the users of these numerical methods, who may have chosen to use a 1st or 2nd order sensitivity analysis and wish to know which is more accurate/fast/accessible.

We have added notes on the differentiation between accuracy and prediction accuracy to the benchmarking section of the manuscript. Additionally, we have added a new subsection to the benchmarking section that addresses specifically the accuracy of the forward, adjoint, and complex perturbation methods (as tested on the non-stiff SIR model and the stiff ROBER model). Because these methods are expected to converge (at least in the non-stiff case) to the true derivative at strict enough tolerances, we show convergence as a function of tolerance for these methods (see the new Figure 6) to demonstrate that they do in fact converge and to measure the distance from the converged value as tolerance is increased. Additionally, these plots provide a way for us to compare the convergence rates of a stiff versus a non-stiff model.

Section 4.1 (the new accuracy section) reproduced below for convenience:

It is important to understand how close computed differential sensitivities are to true differential sensitivities. Unfortunately, the latter are almost always unavailable for ODE models. For the stochastic SIR and branching process models, true sensitivities are well matched by the approximate sensitivities delivered by the complex perturbation methods, provided the complex perturbation is small enough [11]. As a proxy for comparison to true values in ODE models, one can compute the Euclidean distance between sensitivities delivered by the complex perturbation method and the methods relying on the chain rule. In general, we find these distances are very small.

For the forward and adjoint sensitivities of non-stiff ODES such as the SIR and CARRGO models, it is known that as one decrease the tolerance of the underlying solver, the solution and its sensitivities converge to their true values [12]. To demonstrate that the same behavior occurs in our cases, we compute the sensitivities $\frac{\partial S}{\partial \eta}$ of the SIR model and $\frac{\partial x_1}{\partial p_1}$ of the ROBER model at t=1000 using the adjoint, forward, and complex perturbation methods at a variety of tolerances ranging from 1×10^{-2} to 1×10^{-8}.

Figure 6 Title: Convergence of Adjoint, Forward, and Complex Perturbation Methods for Numerical Sensitivities

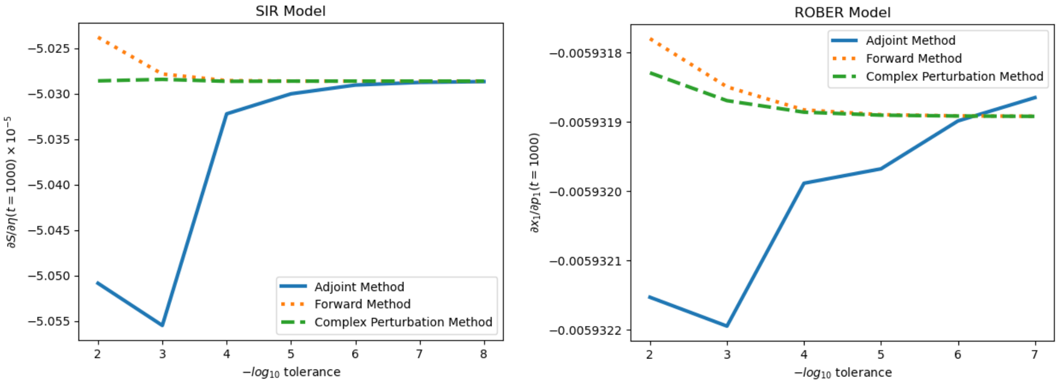

Figure 6 Caption: Convergence plot of the SIR model (top) and ROBER model (bottom) simulated over t=1000 days. For SIR the initial conditions are $S_0 = 3.4 \times 10^8, I_0 = 100$ and the parameters are $\eta = 0.7194, \delta = 0.5025$. For ROBER the initial conditions are $x_1 = 1.0, x_2 = 0.0, x_3 = 0.0$ and the parameters are $p_1 = 4 \times 10^{-2}, p_2 = 3 \times 10^7, p_3 = 1 \times 10^4$. First-order sensitivities are computed via code from this manuscript (complex perturbation method), the ForwardDiff.jl package (forward method) and the Rodas4(autodiff=false) solver under the QuadratureAdjoint(autovec=EnzymeVJP()) sensealg protocol in the DiffEqSensitivities.jl package (adjoint method). All results normalized by the number of time steps included in the simulation.

Figure 6 shows that all three method types (adjoint, forward, and complex perturbation) converge in the non-stiff case (the SIR model). However, each method converges at a different rate and potentially from a different direction. In the case of

> a relatively small, non-stiff model, the complex perturbation method converges more quickly (and at higher tolerance) than the other methods. Notably, when the tolerance for the adjoint method is too weak the method behaves somewhat erratically. This behavior becomes even more pronounced if we consider a stiff ODE model such as ROBER. In this case it is worth noting that the forward and complex perturbation methods converge, albeit under a more stringent tolerance. The adjoint method however struggles to converge for the ROBER model. While the adjoint method should theoretically converge even in the stiff case, it is possible that the tolerance or step size required to do so would render the adjoint method computationally infeasible.

> [11] Squire W, Trapp G. Using Complex Variables to Estimate Derivatives of Real Functions. SIAM Review. 2006.

> [12] Rackauckas C, Ma Y et al. Universal Differential Equations for Scientific Machine Learning.arXiv.2021.

In practice, users tend to face the complex tradeoff between different algorithmic parameters, such as the tolerance of the ODE solver, the step size of the finite-difference method versus computational budget. The provided numerical benchmarks do not seem to give any insights into these important concerns. Please make more explicit what ODE solver was used and what were the values of the error tolerances. Could the users afford to relax some of those error tolerances and still achieve acceptable accuracy? Perhaps a graph showing the dependence between (complex) finite-difference step size and/or ODE solver tolerance versus accuracy will be helpful here.

We have included a subsection that shows solver tolerance versus accuracy that is displayed in Figure 6 (in more detail in response to the comment directly above this one). We have also added captions to all of the figures and tables which include details about solvers and tolerances for the interested reader. Additionally, we have included an additional subsection in the benchmarking section about speed versus accuracy, specifically in the cases of the forward and complex perturbation methods and implemented on the non-stiff SIR and stiff ROBER models. The (new) Figure 7 and subsection 4.2 demonstrate these results, reproduced below for convenience:

> The trade-off between computation speed and accuracy of a solution is relevant to solving ODE systems whether they are stiff or not. Figure 7 displays the time versus error trade-off for both the SIR (non-stiff) and ROBER (stiff) models. In this case, error is calculated as the Euclidean distance between the derivatives calculated at various error tolerances and the derivatives calculated at a strict tolerance of 1×10^{-8} (for the SIR model) and 1×10^{-5} (for the ROBER model). We chose these tolerances as the strictest possible that are numerically realistic for each model. Figure 6 demonstrates that our choices are strict enough for the methods to reach convergence. We display errors versus time in a log-log plot averaged over compartments and parameters and normalized by length of time. We do not include

the adjoint method in this comparison due to its difficulties in convergence and large computational cost.

Figure 7 demonstrates the clear trade-off between speed and accuracy in both the stiff (ROBER) and non-stiff (SIR) cases. In both cases, the forward method can be computed more quickly for equal errors than the complex perturbation method. As expected, the ROBER model has a less steep slope compared to the SIR model, indicating that the returns in accuracy grow more slowly per time invested for a stiff ODE system.

Figure 7 Title: Time vs Error of Forward, and Complex Perturbation Methods for Numerical Sensitivities

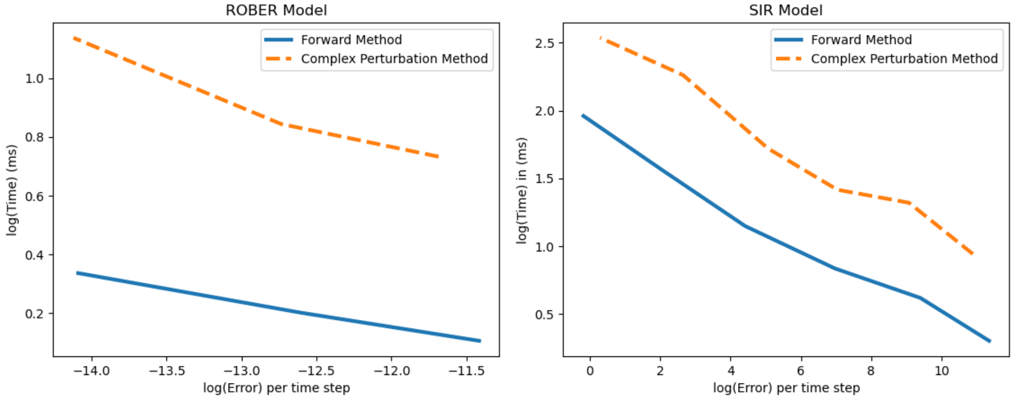

Figure 7 Caption: Time versus error log-log plot of the SIR model (top) and ROBER model (bottom) simulated over t=1000 days. For SIR the initial conditions are $S_0 = 3.4 \times 10^8, I_0 = 100$ and the parameters are $\eta = 0.7194, \delta = 0.5025$. For ROBER the initial conditions are $x_1 = 1.0, x_2 = 0.0, x_3 = 0.0$ and the parameters are $p_1 = 4 \times 10^{-2}, p_2 = 3 \times 10^7, p_3 = 1 \times 10^4$. First-order sensitivities are computed via code from this manuscript (complex perturbation method) and the ForwardDiff.jl package (forward method). Times are median times computed using the Benchmark.jl package, and errors are the Euclidean distance between the solution at the strictest tolerance (10^{-8} for SIR, 10^{-5} for ROBER) and the solution at a variety of tolerances with a maximum of 10^{-2}. All errors normalized by the number of time steps.

Parameter sensitivity analysis is a well-established field that is heavily used in computational biology for the interpretation and design of experiments. Unfortunately, as this paper is written, the introduction and discussion sections do not provide a thorough enough discussion of previous works to set the stage or motivate new efforts this important topic. Instead, the introduction reads more like a collection of anecdotes and generalizations, and the reference list is incredibly short for such a well-studied topic, lacking references from key players in the field such as M. Stumpf, DE Kirschner, F. Doyle, M. Khammash. A more thorough and scientifically documented introduction that better explains the uses for, and limitations of, existing sensitivity analyses would help place the current work in a far more compelling context that would motivate readers to appreciate the importance of the manuscript's results.

We have added a paragraph to the introduction that introduces the topic of parameter estimation, its importance to the field of biological modeling, and the role that differential sensitivity plays in determining parameter estimation. This paragraph serves as a partial literature review of common differential sensitivity methods and as a reminder to the reader that a pertinent context for differential sensitivity is in identifying parameter values. This paragraph is reproduced in this document for convenience, see *Excerpt 1* *(Additionally, parameter estimation for model fitting…)*

The writing of this document does not provide enough discussion of the advantages and limitations of the obtained results, and the manuscript does not sufficiently describe the biological contexts in which these results will, or will not, be important.

We hope that the new format of our benchmarking section allows for a more thorough discussion of the advantages and limitations of the results. Additionally, we have added the following to the discussion section of the manuscript related to when these methods may or may not be appropriate:

> Of the methods mentioned in this text, the adjoint method, forward method, and complex perturbation methods all require that the functions defining a model be differentiable in the underlying parameters. While the complex perturbation method has the additional requirement that these functions be complex analytic, it is the only method explored in this manuscript that can be extended to discrete stochastic models in addition to ODE systems. For the modeler who prefers a one size fits all approach, or who prefers to prioritize ease of implementation, we argue that the complex perturbation method should be the method of choice. In addition to its wide range of applicability, the complex perturbation method can be easily multi-threaded and requires only implementation of the component functions of the model. In contrast to the second-order complex perturbation method, forward differentiation slows dramatically in calculating a Hessian directly. It becomes competitive if one calculates the gradient of the gradient. The gradient of the gradient method is not always available natively and usually must be implemented separately as we have done in the current manuscript. Crucially, implementing a specialized forward mode method was possible due to the underlying automatic differentiation software's flexibility and support for composition.
>
> In situations demanding computational speed, our results suggest that choosing a method more specific to the model may be pertinent. In the case of stochastic models, manually differentiating and applying the chain rule must be balanced against the complex perturbation method, which requires less computation up front but longer processing after the derivatives have been determined. For ODE systems models, forward mode is the most computationally efficient when multichunking is available. If multichunking is not available, then the complex perturbation method has comparable speed to the forward method when run with the same tolerance. In maximizing computational efficiency it is important to note that the use of automatic differentiation tools may require more user input for algorithm selection or multi-threading implementation. Choice of software is critical as well; not all software packages with automatic forward differentiation support chunking as implemented

in the ForwardDiff.jl package and that so dramatically improves the computational efficiency of this method.

There are additional challenges to computing model sensitivity that we do not address. For example, not all models use functions that are differentiable in their parameters. Additionally, models may be differentiable yet extremely stiff, in which case the computational time for all of the sensitivity methods discussed here will suffer disproportionally as the number of parameters grows. Furthermore, assessing global parameter sensitivity is more challenging. It can be attacked by techniques such as Latin square hypercube sampling or Sobel quasi-random sampling, but these become infeasible in high dimensions [13]. Given the availability of appropriate software, differential sensitivity is computationally feasible, even for high-dimensional systems.

In the case of stochastic models, traditional methods require costly and inaccurate simulation over a bundle of parameter values. Differential sensitivity is often out of the question. This limits the ability of researchers to understand a biological system and how it responds to parameter changes. If a system index such as a mean, variance, extinction probability, or extinction time can be computed by a reasonable algorithm, then differential parameter sensitivity analysis can be undertaken. We have indicated in a handful of examples how this can be accomplished.

[13] Qian G, Mahdi A. Sensitivity analysis methods in the biomedical sciences. Mathematical Biosciences. 2020; 323:108306

There are problems with, or missing information in, many figures: Figure 2 lacks a y-axis label. Figure 1 is cropped to obscure the colorbar and is missing a label for the colorbar. Several figure captions are not provided or are not sufficiently descriptive. Fonts on several figures are too small to read for both labels and axes values, and some of the plot lines are too thin to see very well. Please consider carefully to improve the quality and format of the figures -- this could increase the overall quality of the final document…. To improve readability, please provide more information in the captions of the tables, explaining how the methods are mapped to the numerical schemes in the main text.

We have reformatted all figures using the PyPlot package with custom font sizes, colors, linestyles, and formatted axes. We have broken up tables into more manageable pieces that match with the benchmarking subsections. Additionally, we have added captions to all figures and tables with detailed descriptions of how we arrived at our results and any new language or abbreviations that are used.

The discussion of the advantage of multithreading on the MCC model (Lines 664-665 main text) is not convincing and seems to contradict the CPU times reported in the last sub-table of Table 2. In particular, the multi-threaded versions of both first and second-order methods seem to improve only slightly for the case t_end=10 and have mixed outcomes for t_end=100 and t_end=1000. Moreover, the specifics of how multithreading is

accomplished are not sufficiently clear in the manuscript… In most cases shown in Table 2, it appears that the "Multi-Threaded Analytic" version does not improve, and even slightly worsen, computational time compared to "Analytic." If the computations of the gradient entries are done in parallel, I would expect something close to linear scalability. Is there any explanation for this?

While linear scalability of multi-threading methods is the ideal scenario, unfortunately that is not the case in practice. When we explicitly add multi-threading to a method, we are limiting the utility of any implicit multi-threading in the method (for example, in the internal reliance on BLAS operations). Additionally, in any programming language, including Julia, there are significant start-up costs to starting up a thread. These start-up costs may overshadow the benefits of multithreading if the amount of computation per thread is not high enough. Thus, in smaller models (such as the SIR), it is not expected for the multi-threaded methods to outperform their single threaded counterparts. Even in larger models, while we do observe an improvement in computation time, it is not expected for the addition of multiple threads to cause a linear improvement. Additionally, garbage collection is not multi-threaded in Julia. This means that if a method requires a larger number of memory allocations, then it will be slowed down more by multi-threading. This can help account for the difference in improvement in multi-threading in the forward method versus the complex perturbation method. We have added a similar explanation of these issues to the manuscript here (reproduced below for convenience):

> While multi-threading usually decreases computational time for both first-order and second-order methods, it does not decrease computational time by as wide of a margin as expected. Many of the solver methods for stiff ODEs rely on BLAS operations that are already internally optimized by running on multiple threads. Explicitly multi-threading sensitivity methods therefore restricts the number of threads available for BLAS operations,
> adversely affecting their performance. In addition to the reduced efficiency of BLAS operations, multi-threading incurs a start-up cost for each thread. These start-up costs may overshadow the benefits of multi-threading if the amount of computation per thread is not high enough. Multi-threaded methods require more allocations than other methods, and thus require more garbage collection. While time spent on garbage collection varies, we find that garbage collection can take over twice as much computational time in multi-threaded methods than in their single-threaded counterparts. Thus, multi-threading can only really start to improve computational efficiency when these additional costs are small compared to the cost of each computation. Multi-threading may even be less efficient in some cases.

Multithreading is mentioned in the manuscript, but the example Julia routines did not seem to have any thread directives. How much complexity would be added to the code if we wish to implement the multi-threaded versions of the methods?... The insertion of the Julia codes in the main text is somewhat distracting, especially for readers who may be more experienced in other coding languages. We would recommend moving the specific Julia codes to appendices and GitHub links, and then replace the algorithms in the main text with language-agnostic pseudo code.

Thank you for this suggestion. In fact, adding multi-threading for the complex perturbation method only requires one additional line of code. We have added multi-threading into the existing Julia routines for calculating both gradients and hessians. In order to highlight the ease of use of the complex perturbation method, we have elected to keep our code in the main body of the manuscript. Since the code excerpts are short, we hope that they serve as illustration to the user that this method can be implemented as simply as the base model itself. Additionally, using the Julia programming language allows us to display the ease of multi-threading these methods specifically. Since programming languages differ drastically with respect to how multi-threading is implemented, it would be nearly impossible to highlight this benefit using pseudo-code.

Please provide more details on how parallelism is done for the implementations. For example, with the complex-analytic method, is each function evaluation distributed among threads, but is the computation of the ODE at each parameter perturbation done sequentially?

We have added the following paragraphs to the manuscript in the benchmarking section when we compare computation time and introduce multi-threading and multi-chunking. For more information on the difference between multi-threading and multi-chunking, please see our response to the next comment. Multi-threading is implemented to calculate the derivatives with respect to different parameters separately. Excerpt included below for convenience:

> Speed is an important attribute of any computational method, especially when it is performed without the benefit of computational clusters or distributed computing resources. Our speed comparisons offer a first look at the efficiency gains possible with multithreading. In implementing multithreading for both the complex perturbation and forward mode methods, we call the Polyester.jl package to compute each partial derivative in a separate thread. All computations were done in Julia version 1.7.1 on a Windows operating system with an Intel(R) Core (TM) i7-8565U CPU.
>
> In addition to multithreading, the forward method as implemented in ForwardDiff.jl package provides the capability of multichunking. This involves splitting the equations in each system into different chunks to be solved separately. While forward methods do benefit from chunking, this tactic is unavailable in many packages outside of ForwardDiff.jl or outside of the Julia language. For biologists who depend on other packages and computer languages, it may be more pertinent to focus on the non-chunked results for the forward method.

In Table 2, it is not clear what the difference between "Parallel FD.jl" and "Multi-Threaded FD.jl" is….In Table 5 of Appendix C, what is the difference between "Parallel FD.jl Gradient" and "Parallel FD.jl Multi-Threaded Gradient"? Are there other parallelisms other than multithreading?

Thank you for pointing out that this is not clear. In these methods, there are two ways that we can split up the computation of derivatives. We can parallelize across parameters (computing derivatives with respect to different parameters separately) or we can break up the systems of equations across compartments (computing derivatives of each state variable separately). Currently, breaking up the computation by compartment requires SIMD via AVX instructions, and thus is only available with forward differentiation, which is implemented in the ForwardDiff.jl package. This utilization of SIMD is what we referred to as "parallel" in the manuscript. Parallelization across parameters is possible in both the forward and complex perturbation methods, but is not currently available in the ForwardDiff.jl package. However, we have implemented parallelization across parameters on our own using the multi-threading capabilities of the Julia programming language and the Polyester.jl package. This parallelization across parameters is what we referred to as "multi-threaded" in the manuscript.

We recognize that this language is confusing. Therefore, we have changed our language in this version of the manuscript. We continue to refer to the parallelization across parameters that we implement as multi-threading; however we refer to the utilization of SIMD across compartments that is native to ForwardDiff.jl as "chunking", since this is how this implementation is referred to in the literature where ForwardDiff.jl is introduced. Furthermore, we now explain "chunking" in detail before we refer to it, as shown in the following new excerpt from the manuscript:

> In this form, forward mode automatic differentiation shares many similarities to the complex perturbation methods described above without the requirement that the extension of $f(x)$ be complex analytic. At every stage of the calculation $f(x)$ must be differentiable, a weaker yet still restrictive assumption. Conveniently, automatic differentiation allows for arbitrarily many derivatives to be calculated simultaneously. By defining higher-dimensional dual numbers that act independently via
>
> $$f\left(a+\sum_i b_i\,\epsilon_i\right)=f(a)+\sum \epsilon_i df(a)b_i$$
>
> one can calculate entire Jacobians in a single function call $f(a+\sum_i e_i\epsilon_i)$. This use of higher-dimensional dual numbers is a practice known as chunking. Chunking reduces the number of primal (non-derivative) calculations required for computing the Jacobian. Because the ForwardDiff.jl package uses chunking by default, we will investigate the extent to which this detail is applicable in biological models.

Appendix B. Mammalian Cell Cycle Model. The authors cite reference 23 as the source of this model. But a quick inspection of the reference reflects a model with 39 ODEs versus the 11 equations in this text. Also, the initial condition values reported in this manuscript are different to the initial conditions reported on the original document. Is the model presented here a simplification of the one presented by Gerard and Goldbetor? If this is the case, why are the authors not explaining these changes to the original model? How are these modifications affecting the original model?

The model that we use is a simplified version of the one presented by Gerard and Goldbetor, as described in BioModels [14]. We have added this citation and clarified where this model comes from in both section 4 of the manuscript (when the model is introduced) and in Appendix B (where the model is explained in more detail).

[14] Malik-Sheriff R S et al. BioModels—15 years of sharing computational models in life science. Nucleic Acids Research. 2020.

In the paragraph starting on line 149, the adjoint variable term λ is not to defined or described to sufficient detail.

Thank you for pointing this out. We have added a more detailed description of λ and the adjoint method in this section of the manuscript by adding the following:

> The adjoint method introduces a Lagrange multiplier $\lambda(\beta)$, numerically solves the ODE system forward in time over $[t_0, t_n]$ then solves the system
> $$d_\beta \lambda(\beta) = d_x f[x(\beta), \beta]\lambda(\beta) + d_\beta g[x(\beta), \beta]$$
> for $\lambda(\beta)$ in reverse time, and finally uses the introduced parameter to compute derivatives via
> $$d_\beta g[x(\beta), \beta] = \int_{t_0}^{t_n} \lambda(t, \beta) d_\beta\, x(t, \beta) dt$$

At or near line 72, please state explicitly that β refers to the model parameters – this will make it clearer to readers later on.

We have replaced this overly mathematical part of the Introduction with the more qualitative description:

> The differential sensitivity of a model quantity is measured by its gradient with respect to the underlying parameters at their estimated values.

Later, when we do reference β, we reference them as "the parameters β" in order to introduce the variable with more clarity.

In section 3.3, how are the second order terms calculated? This does not appear to be included in the preceding Julia codes.

Thank you for pointing out that it is unclear where the reader can find the relevant equations in order to calculate the second order terms. We have added the following paragraph to this manuscript below the second order Taylor approximation to provide the needed clarity (equations 3 and 4 are the complex perturbation methods for computing non-mixed and mixed second derivatives introduced in section 2.3):

> The improved accuracy achieved by including second-order terms often justifies their computation. The complex perturbation method of permits straight-forward

> computation of second derivatives via approximations (3) and (4). The DiffEqSensitivity.jl and ForwardDiff.jl packages implement both forward difference and adjoint methods for computing the second derivatives of differential equation systems.

Some equations are numbered but others are not? The manuscript would be easier to read with all equations given a number…Please hyphenate two-word adjectives before a noun…. various notes about typos.

Thank you for your various suggestions regarding readability. We have added equation numbers to equations that are referenced multiple times and hyphenated two-word adjectives where appropriate. We have also fixed all identified typos, as well as thoroughly read through the manuscript to identify and correct any additional typos not identified by the reviewers.

Reviewer #4

<u>Summary for Reviewer #4</u>

Reviewer 4 was mostly concerned with our lack of stiff models in our benchmarking analysis. We have added the ROBER model (a stiff ODE model) to our benchmarking section, and highlighted the differences and similarities between the behavior of the ROBER model and non-stiff models (specifically, the SIR model). Reviewer 4 would likely be most interested in our reorganization and added experiments in our benchmarking section (section 4), most notably our inclusion of the ROBER model in the convergence accuracy, speed, prediction accuracy, and speed v. accuracy sections.

<u>Responses to Individual Comments for Reviewer #3</u>

My only tiny issue is with the forward difference method mentioned on page 8, no one in their right mind would use that, but the authors do mention the use of the two-point method in the following paragraph.

While the forward difference method mentioned does not have the accuracy advantage of the central difference method, it does require only half the number of computations that the central difference method requires. Thus, in a situation where computation time is of concern, a biologist may choose to use the forward difference method instead of the central difference method. We have the added the following to the manuscript to address this use case:

> However, the central difference method requires twice the number of computations as the forward difference method. Thus, the choice of a difference method depends on prioritization of accuracy versus computational efficiency. In small models, computational efficiency may be less of a priority, in which case central difference methods are preferred.

One question that authors might consider in the discussion is how well these methods perform on really stiff problems and the problem with fast changing variables where the derivatives change rapidly. Differentiation is notoriously bad in such situations, and we,

for example, have to resort to Richardson's extrapolation for tough problems, which is particularly slow.

Thank you for your suggestion to include a stiff model. We have added the ROBER [15] model (a stiff system of ODEs) to all parts of our benchmarking section. In addition to assessing the convergence of differential sensitivity in the ROBER model (see Figure 6 in the manuscript and reproduced for convenience earlier in this manuscript) we have also studied the time versus accuracy tradeoff in the stiff setting (see Figure 7 in the manuscript and reproduced for convenience earlier in this document). In addition, we have added the ROBER model to both our speed and prediction accuracy tables.

You are correct in noting that differentiation by the adjoint method is particularly difficult for stiff problems, as noted in Figure 6, which shows how the adjoint method does not easily converge in the case of stiff ODEs. However, other methods of differentiation (such as the forward and complex perturbation methods shown here) do converge even for stiff problems. While they may require a stricter tolerance to do so as compared with non-stiff problems (in our tests, the forward method converges at a tolerance of 1×10^{-4} for the SIR model and 1×10^{-5} for the ROBER model), and have a less steep time vs accuracy tradeoff curve (as compared to the non-stiff SIR model in Figure 7), the forward and complex step methods do produce results for stiff models. We would not suggest that biologists utilize Richardson's extrapolation in this context; in fact, it has been shown that sources that suggest use of Richardson's extrapolation to solve for derivatives of stiff models [16] may be generally unreliable [17]. Therefore, while stiff models are an important area of research and merit a place in our manuscript, they may not be as intractable as they seem.

[15] Robertson H. The solution of a set of reaction rate equations. Numerical analysis: an introduction. 1966.

[16] Teukolsky, S. A., Press, W. H., Flannery, B. P., Vetterling, W. T. Numerical Recipes in C: The Art of Scientific Computing (3rd Edition). United Kingdom: Cambridge University Press. (2007)

[17] Buerkle, Alex. Why not use Numerical Recipes? University of Wyoming, https://www.uwyo.edu/buerkle/misc/wnotnr.html (last modified 2004).
