# Supplementary material for: Differential methods for assessing sensitivity in biological models
Source: PLoS Comput Biol. 2022 Jun 13;18(6):e1009598. doi: 10.1371/journal.pcbi.1009598 (PMC9232177; doi:10.1371/journal.pcbi.1009598)
Supplement: Attachment — Submitted filename: Letter.pdf [file pcbi.1009598.s007.pdf]

To Whom It May Concern,

The following document contains responses to reviewer comments including excerpts from changes to the manuscript. Reviewers may note that three changes have been made to the manuscript after receiving these reviews –

(1) We have improved our figures such that they meet publication quality standards. Changes include increased font size, grouping of subfigures, and conversion of all figures to a standard PyPlot backend.
(2) We have moved much of the background information and mathematical derivation of sensitivity analysis of linear systems to a new Appendix C: Sensitivity of Linear Systems.
(3) We have included the ForwardDiff.jl package in our benchmarking of branching process methods and added an additional explanation of instances in which automatic differentiation will fail in stochastic models.

Thank you for the opportunity to further enhance our manuscript by implementing the suggestions provided in these reviews.

Sincerely,

Rachel Mester

Reviewer #1:

Reviewer 1 did not request any changes to the manuscript. The authors would like to thank reviewer 1 for the useful feedback and advice offered in round 1 of reviews.

Reviewer #2:

Revision of all figures to meet a publication quality.

We have made improvements to our figures to ensure they reach publication quality. First, we have grouped all subfigures together, to ensure consistency in font size, formatting, and labelling within each figure. Next, we added a PyPlot backend to all figures and increased all font sizes to 11pt, allowing some leeway with the minimum font size for resizing of the figures within the text.

Focus section 4 on ForwardDiff.jl vs complex perturbation, in particular advantages of complex perturbation in branching processes.

In order to facilitate further comparisons between the various sensitivity methods in stochastic models, we have added automatic differentiation from the ForwardDiff.jl package to the benchmarked methods for branching processes. In addition to adding these benchmarks to Table 4, we have added the following to the results section paragraph describing the results for branching processes –

> The derivatives of $\boldsymbol{A}$ are matrix equations, and in this case forward mode differentiation even without chunking performs as well as the complex perturbation method, although it does not scale as well to larger systems ($N = 1000$). However, in the case of the derivatives of $\boldsymbol{e}$, which are calculated using recursion, neither implementation of forward mode differentiation can be computed as quickly as the complex perturbation method, and this difference increases with the size of the system. Other evidence not shown suggests that the complex perturbation method can reliably evaluate sensitivities where solutions depend on linear algebra and/or recurrence relations. In summary, unless derivatives are quite complicated, manual differentiation is generally more computationally efficient than either the complex perturbation method or the forward method. In computing second derivatives, we expect the tables will be turned. To their credit, the forward and complex perturbation methods do not require formulating derivatives analytically in advance and are consequentially easier to implement.

Additionally, while the examples shown in this manuscript allow for both automatic differentiation and the complex perturbation method, there do exist situations in which automatic differentiation will fail. Current automatic differentiation systems, such as PyTorch, Zygote and ForwardDiff, treat generated random numbers as constants. For example, this is done in forward-mode automatic differentiation by defining the dual part of the generated random number to be 0. While one can prove that in many contexts this can give reasonable results, this results in many stochastic models computing a zero derivative. For example, the derivative with respect to p of the sum of 100 Bernoulli random variables with probability p of having the value 1 will be computed as zero when using contemporary automatic differentiation engines. A full investigation of these limitations of automatic differentiation on stochastic models requires its own treatment, and will be addressed in an upcoming manuscript ("Automatic Differentiation of Discrete and Discrete-Continuous Stochastic Programs"). However, we have added a note to the Discussion section of this manuscript that –

> Current automatic differentiation systems, such as PyTorch, Zygote and ForwardDiff, treat generated random numbers as constants, and thus are not reliable methods for use in calculating differential sensitivity of model outcomes that depend on these random variables.

Remove any excess math that does not contribute to the main narrative, use clear and consistent definitions and notations for derivatives and definitions of different types of errors in the introduction sections.

We have moved much of the background information and mathematical derivation from section 2.1 to a new appendix. This streamlines the mathematical material so that only the equations that are directly related to the methods discussed in the manuscript are included in Section 2.

Reviewer #3:

While we agree that it is very helpful to make the Julia code easily available to the reader, the Julia code as currently formatted is still somewhat distracting from the flow of the text. Perhaps this can be improved by placing it in a labeled box - or perhaps it could be presented in a

different font — we recommend working with the journal editors to choose the most appropriate way to present this.

Thank you for the helpful suggestions! We look forward to working with the editors of the journal to present the code snippets in a format consistent with editorial standards.

Some of the figure fonts (especially axes labels and tick numbers) are still too small to read – we really had to zoom in to see these. Please increase to a minimum font size of 9 or 10pt.

We have made improvements to our figures to ensure they reach publication quality. First, we have grouped all subfigures together, to ensure consistency in font size, formatting, and labelling within each figure. Next, we added a PyPlot backend to all figures and increased all font sizes to 11pt, allowing some leeway with the minimum fonts for resizing of the figures within the text.

In Fig. 7 (left), it is not clear why the errors are so large (what do values of 10^10 mean in this context?). We wonder if the Euclidean distance definition for the error is not so informative and perhaps a Euclidean distance for the relative error may be better. I expect that the figure would look the same (except the x-axis numbers would be scaled), so this should not change any of the conclusions.

The size of the error rates in this figure are in part due to the initial compartment sizes, which in these cases range from 0 to 3.4×10^8. Whether or not an adjustment for compartment size would be necessary in an ultimate measure of accuracy would depend upon the specific case for which these sensitivities are intended to be used. Since the goal of this figure is to focus on the qualitative shapes of the graph, we choose to continue to use Euclidean distance, preferring to use a measure familiar to most users in order to avoid obscuring the message of the figure behind yet another mathematical concept.
